# Dynamic and Self-Healable Chitosan/Hyaluronic Acid-Based In Situ-Forming Hydrogels

**DOI:** 10.3390/gels8080477

**Published:** 2022-07-29

**Authors:** Sheila Maiz-Fernández, Leyre Pérez-Álvarez, Unai Silván, José Luis Vilas-Vilela, Senentxu Lanceros-Méndez

**Affiliations:** 1 Macromolecular Chemistry Group (LABQUIMAC), Department of Physical Chemistry, Faculty of Science and Technology, University of the Basque Country, UPV/EHU, Barrio Sarriena, s/n, 48940 Leioa, Spain; sheilamaiz26@gmail.com (S.M.-F.); joseluis.vilas@ehu.es (J.L.V.-V.); 2 BCMaterials, Basque Center for Materials, Applications and Nanostructures, UPV/EHU Science Park, 48940 Leioa, Spain; unai.silvan@bcmaterials.net (U.S.); senentxu.lanceros@bcmaterials.net (S.L.-M.); 3 Ikerbasque, Basque Foundation for Science, 48009 Bilbao, Spain

**Keywords:** *N*-succinyl chitosan, aldehyde hyaluronic acid, hydrogels, dynamic bonds, self-healing

## Abstract

In situ-forming, biodegradable, and self-healing hydrogels, which maintain their integrity after damage, owing to dynamic interactions, are essential biomaterials for bioapplications, such as tissue engineering and drug delivery. This work aims to develop in situ, biodegradable and self-healable hydrogels based on dynamic covalent bonds between *N*-succinyl chitosan (S-CHI) and oxidized aldehyde hyaluronic acid (A-HA). A robust effect of the molar ratio of both S-CHI and A-HA was observed on the swelling, mechanical stability, rheological properties and biodegradation kinetics of these hydrogels, being the stoichiometric ratio that which leads to the lowest swelling factor (×12), highest compression modulus (1.1·10^−3^ MPa), and slowest degradation (9 days). Besides, a rapid (3 s) self-repairing ability was demonstrated in the macro scale as well as by rheology and mechanical tests. Finally, the potential of these biomaterials was evidenced by cytotoxicity essay (>85%).

## 1. Introduction

Hydrogels are hydrophilic three-dimensional polymeric networks with the ability to retain large amounts of water without dissolving and with tunable physical and chemical properties [1]. Hydrogels are promising materials in specific biomedical fields, such as tissue engineering [2], drugs or cells delivery [3], or wound healing [4]. For advanced applications, functional hydrogels with not just the abovementioned properties, but also with self-healing and in situ-forming abilities, have attracted great attention to achieve injectable and long-lasting biomaterials that could fulfill the demanding requirements of personalized medicine.

In situ-forming hydrogels are those capable of responding to external stimuli and become gel quickly due to their characteristic sol-gel transition [5]. These hydrogels have attracted a great deal of interest since their synthesis avoids the use of toxic chemical crosslinking agents. Furthermore, they are easy to administrate and can be injected into the body with minimal invasion. They can be developed from both natural and synthetic polymers. However, the use of natural polymers is highly recommended due to their low cytotoxicity, biodegradability and similarity to natural extracellular matrix (ECM) [6]. In particular, chitosan (CHI) and hyaluronic acid (HA) have gained increasing attention in recent decades because they have demonstrated excellent physico-chemical and biological characteristics for different biomedical applications, including wound regeneration, antibacterial action, tissue engineering, or drug delivery [7,8,9,10].

Chitosan obtained from the partial *N*-deacetylation of chitin is a linear natural polysaccharide composed of repeating units of glucosamine and *N*-acetyl-glucosamine [11]. This polycation has been successfully employed as a biomaterial to develop in situ-forming hydrogels due to its promising features, including its similarity to glycosaminoglycans, versatility, biocompatibility, biodegradability, mucoadhesion and antibacterial activity. Nevertheless, chitosan is generally insoluble in most physiological solvents except in acid media due to its strong intermolecular hydrogen bonding, which considerably limits its application [12]. To overcome this limitation, important efforts have been made to obtain water-soluble chitosan derivatives at physiological pH, most of them via chemical modification, such as *N*-carboxymethyl-chitosan, *O-N*-carboxymethyl-chitosan, *O*-carboxymethyl-chitosan, *N*-sulfate-chitosan, *O*-butyryl-chitosan, *N*-methylene phosphonic chitosan, *N*-trimethyl chitosan, hydroxypropyl chitosan, or *N*-succinyl-chitosan, among others [13]. Indeed, the introduction of succinyl groups at the *N*-position of the glucosamine unit of its backbone leads to the formation of a water-soluble chitosan derivative, *N*-succinyl-chitosan (S-CHI) which has been exploited as an effective way to prepare highly soluble chitosan for biological applications [14].

On the other hand, hyaluronic acid is also a linear natural polysaccharide that is composed of repeating units of *N*-acetyl-glucosamine and *D*-guluronic acid. HA is one of the most hydrophilic molecules found in nature, it is present in all human tissues and is one of the main components of the natural extracellular matrix (ECM). As chitosan, HA also offers good biocompatibility and biodegradability, however, in an enzymatic medium this biodegradation can be excessively rapid, which considerably restricts its applicability [15]. It has been demonstrated that covalent crosslinking of HA is a possible way to overcome this limitation [16]. Typically, chemical crosslinking of polymers takes place by the addition of extra synthetic molecules acting as crosslinking agents of the pristine polysaccharides without any kind of previous functionalization. For example, hydrazine derivatives, 1,4-butanediol diglycidyl ether (BDDE), glutaraldehyde, ethylene sulfide, methacrylic anhydride and divinyl sulfone have been employed to chemically crosslink HA, promoting improved mechanical stability of the biopolymer but leading to toxic effects [17,18]. Due to this, chemical modification, such as oxidation-driven modification reactions, represents a safe alternative to produce polysaccharides with more active groups, favoring in situ chemical crosslinking with other polymers [19]. This is the case of the oxidation of hyaluronic acid to create reactive dialdehyde groups [20]. Following this strategy, hyaluronic acid can be oxidized to form hyaluronic acid dialdehyde (A-HA) because the carbon-carbon bonds of the *cis*-diol groups in the structure cleave to form the new dialdehyde functional groups. Thus, A-HA can act as a crosslinking agent of primary amines (present in proteins and polysaccharides), similarly to glutaraldehyde, and form in situ covalent imine bonds (--CH=N-) by the Schiff’s base reaction in mild conditions [19] and without the necessity of adding any extra chemical crosslinking agents.

Consequently, to overcome the limitations of using external crosslinkers, the development of crosslinker free hydrogels has become an interesting approach to prepare hydrogels for biomedical applications [21]. With this purpose, chitosan and hyaluronic acid have been chemically modified by the pathways described above, to obtain A-HA and S-CHI in situ-forming hydrogels [21]. This is so that the modified A-HA structure can be crosslinked at room temperature when it is mixed with water-soluble S-CHI, leading to in situ hydrogel formation at physiological conditions [20]. A considerable number of research groups have used this strategy to generate crosslinker-free hydrogels of chitosan or hyaluronic acid, showing improved properties thanks to the formation of imine bonds [22]. Oxidized hyaluronic acid has been also used as crosslinker for other polysaccharides such as carboxymethyl chitosan to prevent postoperative adhesion or for regeneration of abdominal tissue [15,23]. Similarly, *N*-succinyl chitosan has also been covalently crosslinked by the Schiff’s base reaction with different polysaccharides, such as oxidized alginate [24,25] or oxidized dextran [26]. All these studies have shown that hydrogels formed by imine linkages of oxidized polysaccharides are promising materials, especially in postoperative stages for adhesions prevention. However, few studies have investigated the combination of both S-CHI and A-HA. This is the case of Rafailevna et al. [27] who optimized the conditions to obtain covalently crosslinked S-CHI/A-HA hydrogels by simple mixing, according to the polymer concentration in solution and the molecular weights of the polymers. Interestingly, this work showed that the rheological properties of prepared hydrogels could be modulated by varying experimental conditions. Furthermore, Zhu et al. [20] used S-CHI/A-HA hydrogel as a support for embedding micelles loaded with insulin and growth factors. Thanks to the good cellular compatibility of these hydrogels, the possibility of using them as wound-healing materials was confirmed in vivo. However, none of the aforementioned works studied the possible self-healing capacity of these hydrogels that can be expected as a result of the dynamic nature of the promoted imine bonds.

Self-healing is the intrinsic ability of some materials to repair themselves after being damaged. This ability found in living tissues is given by reversible interactions capable of being autonomously re-established after being broken. This is the case for a wide variety of dynamic covalent bonds, including phenylboronic ester bonds [28], dynamic imine bonds [29], disulfide bonds [30], acylhydrazones bonds [31] and Diels-Alder reactions [32], or physical interactions such as hydrogen bonding [32], hydrophobic interaction [33], host-guest interaction [34], and ionic interaction [35]. A dynamic covalent bonding approach offers the mechanical stability characteristic of chemical bonds together with the reversible nature of physical interactions [36]. Imines, also called Schiff’s bases, correspond to -C=N groups that are dynamic covalent bonds prepared by the condensation between poly-diamines and poly-dialdehydes leading to 3D-crosslinked networks. Dynamic imine bonds have been exploited for the development of quickly-gelled hydrogels with proven self-healing ability [37]. This is the case of gelatin-based self-healable hydrogels formed by a Schiff-base reaction between the amine groups present in gelatin backbone and aldehyde moieties present in poly(ethylene glycol) di-benzaldehyde. This hydrogel is formed in no more than 20 s and presents the ability to quickly self-repair in 10 min without the need to add any external chemical or physical stimuli [38]. Another good example is the hydrogel system developed by Zhang et al. [39] based on chitosan and dibenzaldehyde-terminated telechelic poly(ethylene glycol). This hydrogel was synthesized under mild conditions and the reaction took place quickly in less than a minute. The dynamic equilibrium between the amine and aldehyde reactants and the Schiff-base linkages confers to the hydrogel a good ability to self-repair (2 h) and to respond to different biochemicals, such as amino acids or B6 vitamin derivatives together with pH changes. 

In the light of the above described results, this work reports on self-healing hydrogels based on S-CHI/A-HA interactions with modular degradation, swelling, mechanical and rheological properties [40]. For that, chitosan and hyaluronic acid were successfully modified introducing succinyl and aldehyde moieties, respectively, along polysaccharides backbone, as verified by ^1^H-NMR, FTIR and colorimetric methods. Subsequently, stable hydrogels were prepared by simple mixture of the modified polysaccharides varying polysaccharides molar ratio, and crosslinking was confirmed by FTIR analyses. This work demonstrates that polysaccharides’ ratio variation provides tailored degradation grades, swelling ability and mechanical and rheological properties that are of great importance for tissue engineering and regeneration applications. In addition, it was herein hypothesized for the first time the self-healing capacity of selected S-CHI/A-HA hydrogels, which increases their interest as functional biomaterials. Finally, their potential to be applied in the biomedical area is confirmed through cytotoxicity tests.

## 2. Materials and Methods

### 2.1. Materials

Hyaluronic acid (Contipro, high molecular weight) and chitosan from crab shells (Sigma Aldrich, highly viscous, St. Louis, MO, USA,) were used for the development of the dynamic covalent hydrogels. The deacetylation degree of chitosan was measured by ^1^H-NMR (85%). Gel permeation chromatography (GPC) was employed for the determination of the average molecular weights of HA and CHI polymers, being 2.1 × 10^6^ ± 1.01 × 10^5^ g/mol and 8.7 × 10^5^ ± 4.0 × 10^4^ g/mol, respectively. The acetic acid (for analysis, 99.8%), methanol (pure, pharma grade), ethanol absolute (for analysis), succinic anhydride (≥99%), sodium (meta)periodate (≥99%), ethylene glycol (≥99%), sodium phosphate monobasic (≥99%), sodium hydroxide (pure, pharma grade), ninhydrin reagent (2% solution), deuterium oxide (99.9% atom D), and acetic acid-d_4_ (≥99.5% atom D) were purchased from Sigma Aldrich.

### 2.2. Modification of Chitosan

Chitosan was modified so as to obtain water-soluble *N*-succinyl-chitosan. For this, S-CHI was synthesized according to a reported procedure [16] with slight modifications. Briefly, 0.5 g of pristine chitosan was dissolved in acetic acid 0.5 % (*v*/*v*) and subsequently, 130 mL of methanol was added to dilute the solution under constant stirring at room temperature. Then, 1.5 g of succinic anhydride was dissolved in 30 mL of methanol and added to the solution. The mixture was left 24 h under the conditions mentioned above and once the time had elapsed, it was precipitated raising the pH to 6–7. The precipitate was re-dissolved in H_2_O and purified under exhaustive dialysis for 3 days. Then, the purified product was lyophilized (−50 °C, 0.1 mBar) and stored at 4 °C. The succinylation degree (SD) of S-CS was determined by ninhydrin assay and nuclear magnetic resonance (^1^H-NMR).

### 2.3. Ninhydrin Assay

For the quantification of the succinylation degree of the modified chitosan, the ninhydrin assay colorimetric method was used according to Curotto et al. [41] with slight modifications in order to determine the remaining free amino groups of modified S-CHI. For this aim, a blank solution of chitosan (0.05% *w*/*w*) was prepared dissolving the polysacchayde in an acetic/acetate buffer solution (pH 5.2, [HAc] = 0.175 M and [NaOH] = 0.128 M). Then, for the preparation of each sample, aliquots between 0.5–1.5 mL were taken and mixed with 1 mL of ninhydrin for 30 min at 110 °C under vigorous stirring. Subsequently, the solutions were cooled to room temperature and diluted with 50 mL of 1:1 molar ratio of ethanol:water. Finally, the absorbance was measured at 570 nm in a Cintra 303 spectrophotometer. The obtained calibration curve is: y = 8.106x + 0.0992; R^2^ = 0.9932. The remaining amounts of primary amine groups of modified *N*-succinyl-chitosan were quantified by using the obtained standard calibration curve at 570 nm and following the procedure described previously [42]. For this, from a solution of 20 mg/mL of S-CHI, small amounts around 0.15 g were mixed with ninhydrin and the same procedure as explained above was followed.

### 2.4. Modification of Hyaluronic Acid

Hyaluronic acid was oxidized to include aldehyde groups in its backbone. For this aim, A-HA was synthesized according to Tan et al. [16]. Briefly, 1.0 g HA was dissolved in 100 mL of distilled water and 5 mL of aqueous solution of sodium periodate 0.5 M was added drop by drop. Subsequently, the reaction took place under constant stirring for 2 h at room temperature and in the dark. Finally, in order to inactivate any unreacted periodate, 1 mL of ethylene glycol was added and the reaction was stirred in the dark at room temperature for 1 h. The solution was purified by exhaustive dialysis against H_2_O for 3 days and the purified product was lyophilized (−50 °C, 0.1 mBar) and stored at 4 °C. The degree of oxidation was determined by the quantification of aldehyde groups by nuclear magnetic resonance (^1^H-NMR).

### 2.5. Synthesis of S-CHI/A-HA Based Hydrogel

The modified polymers were dissolved separately in PBS solution at a concentration of 20 mg/mL. S-CHI and A-HA were then thoroughly mixed by constant stirring (manual or vortex ~1–2 min) at different ratios at room temperature, remaining then without agitation for 15 min in order to achieve the defined shape of the vessel. Magnetic agitation can avoid hydrogel forming. The compositions (molar equivalents) of the gels based on S-CHI:A-HA are 1:9, 3:7, 5:5, 7:3 and 9:1.

### 2.6. Physico-Chemical Characterization

#### 2.6.1. Nuclear Magnetic Resonance (^1^H-NMR)

^1^H-NMR spectroscopy was employed to corroborate the modification reactions of both chitosan and hyaluronic acid, as well as to determine the degree of succinylation and oxidation of both syntheses. Briefly, ^1^H-NMR spectra were taken in D_2_O on a Bruker Avance 500 MHz spectrometer at 25 °C employing a polymer concentration of 1.5% *w*/*w*. For the quantification of the modification of chitosan (succinylation degree, SD) the ratio of the integral area of the protons of the pristine chitosan cycle and the peaks corresponding to succinic anhydride groups at 2.4 ppm were taken into account, according to Equation (1):

SD (%) = ((Integral of *N*-succinyl protons at 2.4 ppm)/4)/((Integral of chitosan H2–H6 at 2.8–3.9 ppm)/6) × 100 (1)

For the quantification of the oxidation of hyaluronic acid (OD; oxidation degree), the ratio of the area of the integral of the signal of the protons of the methyl group of pristine hyaluronic acid and that of the peaks of anomeric protons in the new structure at 4.8 and 5.1 ppm were taken into account, according to Equation (2):

OD (%) = ((Integral of anomeric protons 4.8–5.1 ppm)/1)/((Integral of acetyl group protons at 1.9)/3) × 100(2)

#### 2.6.2. Fourier-Transform Infrared Spectroscopy (FTIR)

Nicolet Nexus FTIR spectrometer (Thermo Scientific, Loughborough, UK) was used in order to study the formation of new covalent bonds in S-CHI/A-HA hydrogels together with the previous modification of CHI and HA polysaccharides. The experiment corresponding to pristine CHI and HA was carried out by KBr pellets and those of modified polysaccharides as well as hydrogels by FTIR-ATR, at a resolution of 4 cm^−1^ and 32 scans per spectrum.

#### 2.6.3. In Vitro Swelling

The capability to absorb water of the prepared hydrogels was determined as follows. Prepared hydrogels were lyophilized at −50 °C and 0.1 mBar. Subsequently, they were immersed in phosphate buffer solution (PBS) (pH = 7.4) at 37 °C in order to imitate physiological conditions. The swelling ratio was measured over time and the swelling factor was calculated according to Equation (3):

Swelling factor= (W_s_ − W_d_)/W_d_ (3)

where, W_s_ and W_d_ are the weights of the swollen and dried hydrogels, respectively. Three samples were analyzed for each composition, and the averages and standard deviations were displayed.

#### 2.6.4. Rheology

The dynamic rheological behavior of S-CHI/A-HA cylindrical hydrogels was evaluated by oscillatory rheometry. The evaluation of the storage (G′) and loss modulus (G″) was analysed by frequency sweep measurements performed in an Advanced Rheometric Expansion System (ARES), using parallel plate geometry (25 mm of diameter) with a gap distance of 1.5 mm at 25 °C. First, a shear strain sweep was recorded to determine the linear viscoelastic region. Then, frequency sweep measurements were carried out from 0.1 to 500 rad·s^−1^ at a fixed strain of 1%. Both strain and frequency sweeps were measured in triplicate.

Self-healing ability of the hydrogels with a composition of S-CHI/A-HA 3:7, 5:5 and 7:3 was analyzed, measuring the rheological properties of the hydrogels along three cut-repair cycles, according to the procedure described above. Furthermore, this ability was also demonstrated by a variation of the storage modulus and the loss modulus when different values of strain were applied at room temperature. For this, hydrogels were sequentially kept (60 s) under their deformation limit (0.1%) and over their deformation limit (80%).

#### 2.6.5. Compressive Stress/Strain Test

To evaluate the mechanical stability of the prepared S-CHI/A-HA hydrogels, compression tests were performed with a Metrotec FTM-50 (20 N load cell) at room temperature and a deformation rate of 1 mm/min. The effect of hydrogel composition together with its self-healing capacity was tested. Compression tests of healed samples were carried out in a perpendicular direction to the cut surface. Young moduli average of each hydrogel were calculated in the corresponding linear region, 40–60% strain range, taking into consideration at least three replicates.

### 2.7. Functional Characterization

#### 2.7.1. In Vitro Biodegradation

Freshly prepared S-CHI/A-HA hydrogels were immersed in phosphate-buffered saline solution (PBS) at pH 7.4 at 37 °C. The in vitro degradation was followed gravimetrically over time according to Equation (4) (three replicates):

Mass loss (%) = 100 − ((W_0_ − W_t_)/W_0_) × 100 (4)

where W_0_ is the weight of the swollen hydrogel at initial time and W_t_ at a specific time. For each composition three replicates were studied.

#### 2.7.2. In Vitro Cytotoxicity Essay

To assess the biocompatibility of the hydrogels, a live/dead assay was performed. Briefly, mouse embryonic fibroblasts (MEFs) were plated in a 24-well plate containing 2.10^5^ cells/well and cultured in standard conditions (37 °C and 5% CO_2_) in complete medium (DMEM with 10% fetal bovine serum and 1% penicillin). Small fragments of S-CHI/A-HA hydrogels (5–10 mg) were washed with PBS and complete medium, dried and sterilized (UV light for 1 h). 24 h later, hydrogels were taken and washed cell cultures (PBS) were stained with Calcein-AM (2 µM), ethidium homodimer (EthD-1, 4 µM), and NucBlue (Thermo Fisher R37605, 2 drops/well). Cell viability was determined from fluorescence images of cells (Leica DMi8 fluorescence microscope) by the ratio between red-stained nuclei and the total number of cells (blue-stained). For this, Fiji software [43] was employed and five images were considered for three independent samples.

## 3. Results and Discussion

### 3.1. Synthesis of Polysaccharide Derivatives

**Figure 1:**
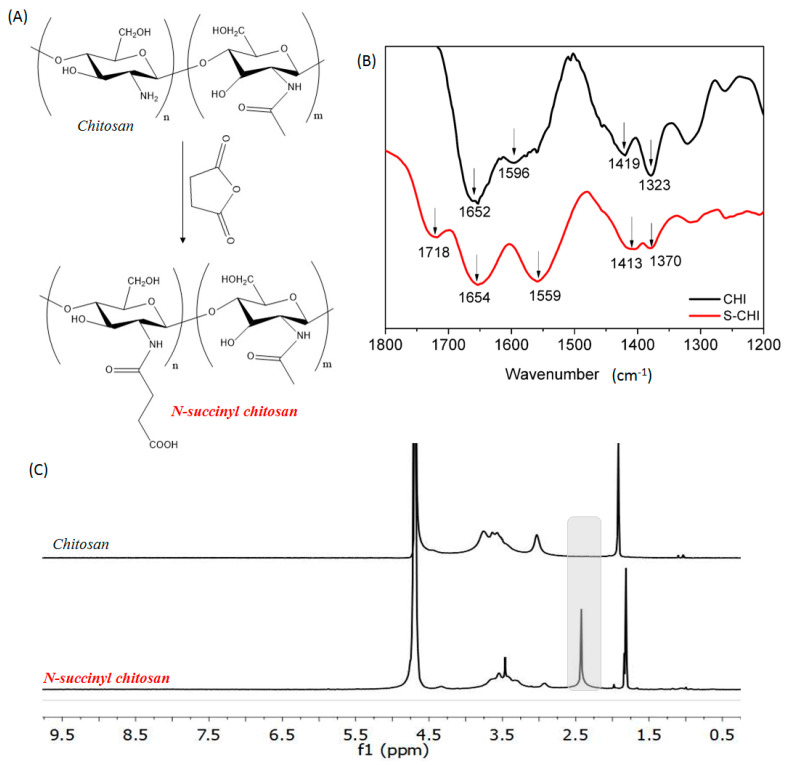
(**A**) Chemical structures of chitosan and *N*-succinyl-chitosan and their (**B**) FTIR and (**C**) ^1^H-NMR spectra.

Chitosan is a natural polysaccharide widely used for the manufacture of hydrogels for tissue engineering applications due to its properties, such as mucoadhesion, biodegradability, and biocompatibility [5]. The presence of primary amino groups in its structure that are highly reactive and accessible confers on chitosan its characteristic simple chemical modification. *N*-succinyl-chitosan has been obtained via ring opening reaction by the introduction of succinyl groups into primary amino groups of the glucosamine units (Figure 1A). The modification reaction between chitosan and succinic anhydride consists of the condensation of the primary amine groups of chitosan and the carbonyl group of the succinic anhydride [16]. This condensation leads to the formation of a new covalent amidic bond as a consequence of the opening of the anhydride group. The presence of this new bond as well as the other characteristic bands of chitosan can be seen in Figure 1B, in which FTIR spectra of both pristine and modified chitosan are presented. The FTIR spectrum of chitosan shows the characteristic absorption bands at 1652 and 1596 cm^−1^, which correspond to the stretching of amide I and amide II bands, respectively. In addition, at a 1419 and 1323 cm^−1^ wavenumbers the twisting of CH_2_ and C-N stretching can also be observed which is in agreement with the literature [44]. The overlaying of both unmodified and modified chitosan spectra shows that the latter, in addition to the above characteristic absorption bands, displays two absorption bands at 1718 and 1413 cm^−1^, indicating the asymmetric and symmetric stretching of the added new carboxylate. Thus, the presence of those absorption bands reveals that the modification reaction of chitosan takes place successfully, which is in line with previously reported works [44,45,46].

Furthermore, so as to corroborate the modification reaction as well as to determine the succinylation degree of *N*-succinyl-chitosan, ^1^H-NMR spectroscopy was employed. The ^1^H-NMR spectra of both pristine chitosan and *N*-succinyl-chitosan are illustrated in Figure 1C. Although both spectra are similar, in the modified chitosan spectrum a new peak appears around 2.4 ppm, which indicates the presence of the succinyl group (–CH_2_–CH_2_–) [47,48,49]. Finally, consistent with the integration of the peak of succinyl group (2.4 ppm, 4 protons) and that of H2-H6 protons of *D*-glucosamine unit (2.8–3.9 ppm, 6 protons) [49], the succinylation degree of modified chitosan was determined to be 57%. Finally, the succinylation degree was also quantified by the ninhydrin assay in which 64 ± 5% was obtained. This value can be considered similar to that obtained by ^1^H-NMR, taking into consideration the differences between both methods.

**Figure 2:**
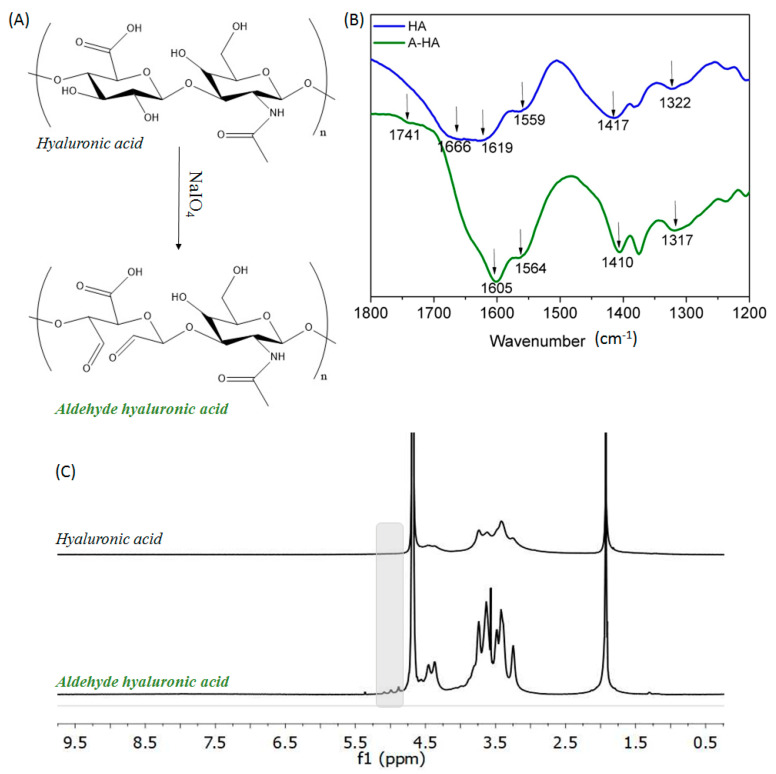
(**A**) Chemical structures of hyaluronic acid and oxidized hyaluronic acid dialdehyde and their (**B**) FTIR and (**C**) ^1^H-NMR spectra.

Hyaluronic acid was also modified by oxidation reaction using sodium periodate, which is one of the most promising hydroxyl-group-mediated modification methods [19]. Periodate sodium salt oxidized vicinal hydroxyl moieties that are present in the repetitive unit of polysaccharide backbone (Figure 2A). This reaction formed two aldehyde groups at the oxidized carbons of the monomeric sugar units, as a consequence of the opening of the sugar ring due to the rupture of the C-C bonds [19]. It is important to highlight that the new aldehyde groups created after the oxidation process might also have reacted with the remaining hydroxyl groups that were present in the hyaluronic acid backbone, leading to the formation of cyclic hemiacetals groups. Figure 2B presents the FTIR spectra of both pristine and modified hyaluronic acid. In the case of unmodified hyaluronic acid, the characteristics absorption bands are found at 1666 and 1659 cm^−1^ corresponding to the C-N stretching of amide I and amide II, respectively. Moreover, at 1619, 1417 and 1322 cm^−1^ the stretching of asymmetric and symmetric bands of carboxylate groups as well as the stretching of C-N can be distinguished [50,51]. Regarding the infrared spectra of oxidized hyaluronic acid, no relevant differences can be highlighted, as is reported in the literature [16]. However at 1741 cm^−1^, a shoulder appeared that corresponded to C=O symmetric vibration of aldehyde moiety, which validated the modification reaction [45,51].

Furthermore, as in the case of chitosan, ^1^H-NMR spectroscopy corroborates the oxidation reaction and was used to determine the oxidation degree. The ^1^H-NMR spectra of both pristine hyaluronic acid and aldehyde hyaluronic acid are illustrated in Figure 2C, where it can be observed next to the characteristic signs of the sugar rings (3.05–4.0 ppm), three low intensity peaks (4.8–5.1 ppm) that evidence the presence of aldehyde groups present in the oxidized structure of hyaluronic acid [52,53]. Finally, taking into account the integral of the peak of acetyl group (1.9 ppm, 3 protons) and the anomeric proton peak (4.8–5.1 ppm, 1 proton), the oxidation degree of modified hyaluronic acid was 24%, which is similar to that obtained in the reported literature [53].

### 3.2. Physico-Chemical Characterization of S-CHI/A-HA Hydrogels

**Figure 3:**
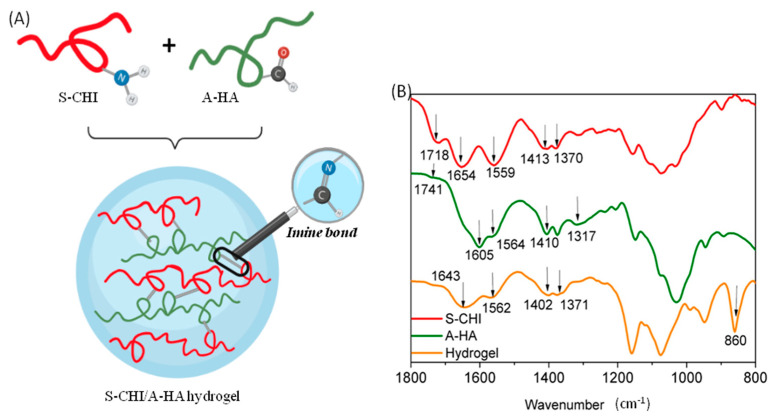
(**A**) Gelation mechanism between *N*-succinyl chitosan and aldehyde hyaluronic acid by the Schiff’s base reaction. (**B**) FTIR spectra of the modified polysaccharides and the corresponding 5:5 S-CHI/A:HA hydrogel.

In situ-forming hydrogels are promising materials for biomedical applications since they provide the possibility of being used as local carriers for cell delivery or due to their potential to promote direct growth or encapsulation and delivery of cells during tissue regeneration, among others. In this study, in situ S-CHI/A-HA hydrogels were successfully developed. The aldehyde moieties present in the A-HA backbone have a positively charged carbon which is highly sensitive to a nucleophilic attack. Furthermore, it can react with many different polar groups or with other polymers containing amine groups, such as *N*-succinyl-chitosan which acts as the Schiff’s base [54]. Thus, the formation of S-CHI/A-HA hydrogel was achieved by a gelation mechanism given by the formation of imine bond (-CH=N-) between the aldehyde moiety of hyaluronic acid and the primary residual amine group of modified chitosan (Figure 3A).

FTIR spectrum (Figure 3B) of the hydrogels suggests that the Schiff’s base reaction between modified polysaccharides takes place successfully. The overlaying of the infrared spectra of the hydrogel with those of the modified polymers leaves evidence of some new vibration bands. This is, for example, the case of the band that appears at 1643 cm^−1^, which according to literature [55] corresponds to the formation of the new covalent imine bonds (-C=N-). In addition, an intense peak appears at 860 cm^−1^, which indicates the presence of hemiacetal groups formed by the nucleophilic addition of the alcohol to the carbonyl group [56]. The obtained data are in good agreement with the literature and corroborate the crosslinking reaction between the primary amines of *N*-succinyl-chitosan and the aldehydes of the oxidized hyaluronic. 

**Figure 4:**
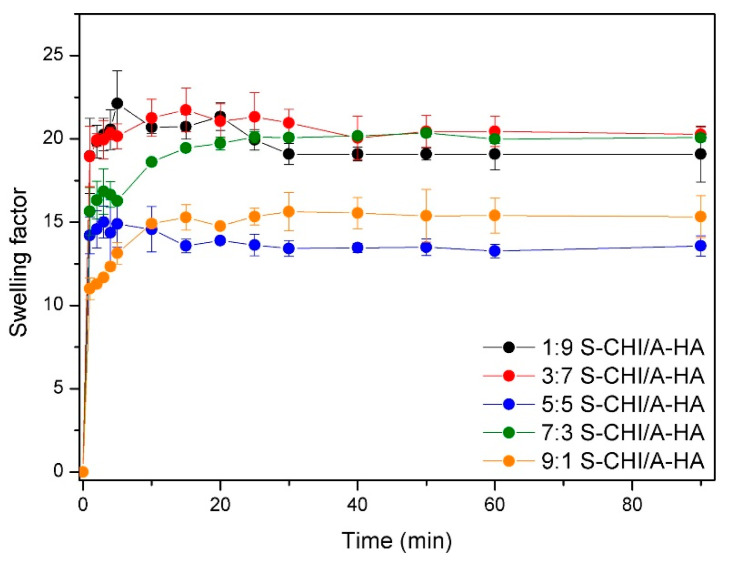
Swelling factors of the different hydrogels.

*N*-succinyl-chitosan and aldehyde hyaluronic are hydrophilic polymers, and thus, the swelling behavior of the different hydrogels was studied and presented in Figure 4. Obtained results shows that the water uptake is higher for the samples with the smallest S-CHI content. Indeed, lower chitosan content implies subsequently, high hyaluronic acid content that potentially favors higher water uptakes due to the highly hydrating ability of HA biopolymer. Interestingly, the lowest swellings were measured for the hydrogels with 5:5 and 9:1 S-CHI:A-HA feed content. This can be ascribed to the competition of the favoring hydration effect of HA with the opposing effect of CHI. This swelling decreasing effect of CHI can be explained by its higher mobility, due to its lower molecular weight, which enhances reaction with A-HA, and thus crosslinking, as well as to chitosan characteristic trend to form strong intra/intermolecular H-bonds at physiological pH, leading to additional physical crosslinking [57]. 

**Figure 5:**
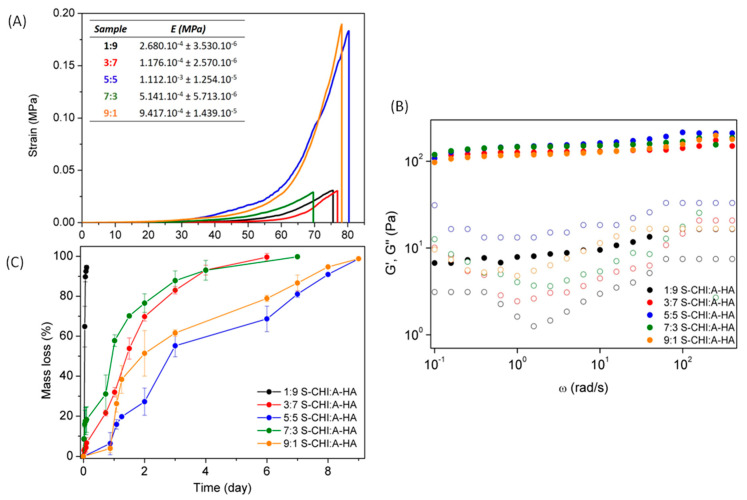
(**A**) Compressive tests, (**B**) Rheological frequency sweep measurements (filled circles G′ and open circles G″) and (**C**) Degradation kinetics of the S-CHI/A-HA hydrogels with different polysaccharide content.

Unconfined compression tests of prepared different hydrogels were also carried out and presented in Figure 5A.

Overall, it can be observed that the maximum deformation until break does not depend on crosslinking density, and all hydrogels achieved deformations of around 75%. However, the strain required to reach this maximum deformation (70–80%) shows a direct dependence on the composition of the material, as previously was observed in swelling data, due to the effect on the crosslinking density of the networks. That is, hydrogels with greater swelling, due to a low CHI content and high HA content (1:9, 3:7 and 7:3, S-CHI: AHA) are deformed more easily. Meanwhile, hydrogels with lower water swelling ability (5:5 and 9:1) display high crosslinking density and therefore are the ones that require the greatest efforts to be deformed and, furthermore, show the highest Young’s moduli. It is important to highlight that 5:5 and 9:1 S-CHI:A-HA hydrogels show similar mechanical behavior, which can be ascribed to their equal (and lower) water uptake, despite covalent crosslinking density is significantly lower for the 9:1 S-CHI:A-HA hydrogels. Thus, it seems that swelling of 9:1 hydrogels is mainly governed by CHI-CHI physical interactions. This behavior reveals that, as was previously observed on swelling, there exists a compromise between the relative feed content of S-CHI and A-HA that promotes an opposite effect. In this sense, 5:5 hydrogels seem to balance both effects, although hydrogels with opposite stoichiometry, such as 1:9 and 9:1, or 3:7 and 7:3 hydrogels which could be expected to behave similarly, show a clearly different behavior according to the nature of the biopolymer in excess. That is, CHI excess seems to favor intramolecular H-bonds leading to a larger number of crosslinking points that results in improved mechanical stability and lower swelling, unlike HA that enhances water uptake and poorer mechanical properties.

On the other hand, the formation of the hydrogels was also confirmed by rheology, since in all cases the storage modulus (G′) was greater than the loss modulus (G″) (Figure 5B), indicating that the elastic properties predominate over viscous ones. In brief, it can be observed that practically all hydrogels showed similar storage modulus values, with the exception of the S-CHI/A-HA 1:9 hydrogel, which was the hydrogel with the lowest crosslinking density.

### 3.3. In Vitro Hydrolytic and Enzymatic Degradation

The biodegradability of hydrogels is a key characteristic since it can considerably limit their application. The weight loss profile of these materials represents a critical issue, in particular in applications that require tailor-made profiles [58].

Accordingly, the dissociation of the synthesized S-CHI/A-HA hydrogels was studied at 37 °C and in physiological pH (Figure 5C). Figure 5C shows that the polysaccharides composition has a strong influence on the physical degradation kinetics of the hydrogels. Hydrogels based on 1:9 S-CHI:A-HA presented the faster degradation profile, achieving 100% of the mass loss in less than a day due to their low crosslinking density. This behavior was in agreement with the reported literature [16] and with above described rheological and swelling. On the other hand, 3:7 and 7:3 S-CHI:A-HA hydrogels, which presented a higher molar ratio between two polysaccharides, also showed greater resistance to degradation, as it took 7–8 days for the material to disintegrate completely. Finally, the 9:1 and 5:5 S-CHI:A-HA hydrogels were the ones with the greatest resistance to disintegration. On the one hand, 5:5 S-CHI:A-HA hydrogels were the ones with the highest molar ratio between two polymers and, therefore, those with the greatest facility for establishing dynamic covalent bonds. In addition, as previously verified, these hydrogels together with the 9:1 S-CHI:A-HA hydrogels presented the highest mechanical stability due to a high crosslinking density, which also led to a slower disintegration that exceeded 9 days. This behavior of 9:1 S-CHI:A-HA hydrogels, as has been mentioned, is related to the ability of N-succinyl-chitosan to form intramolecular hydrogen bonds at pH values higher than its pKa (pH > 6.5), which typically is known as the fundament of chitosan precipitation at neutral pH [59].

### 3.4. In Vitro Cytotoxicity Assay

**Figure 6:**
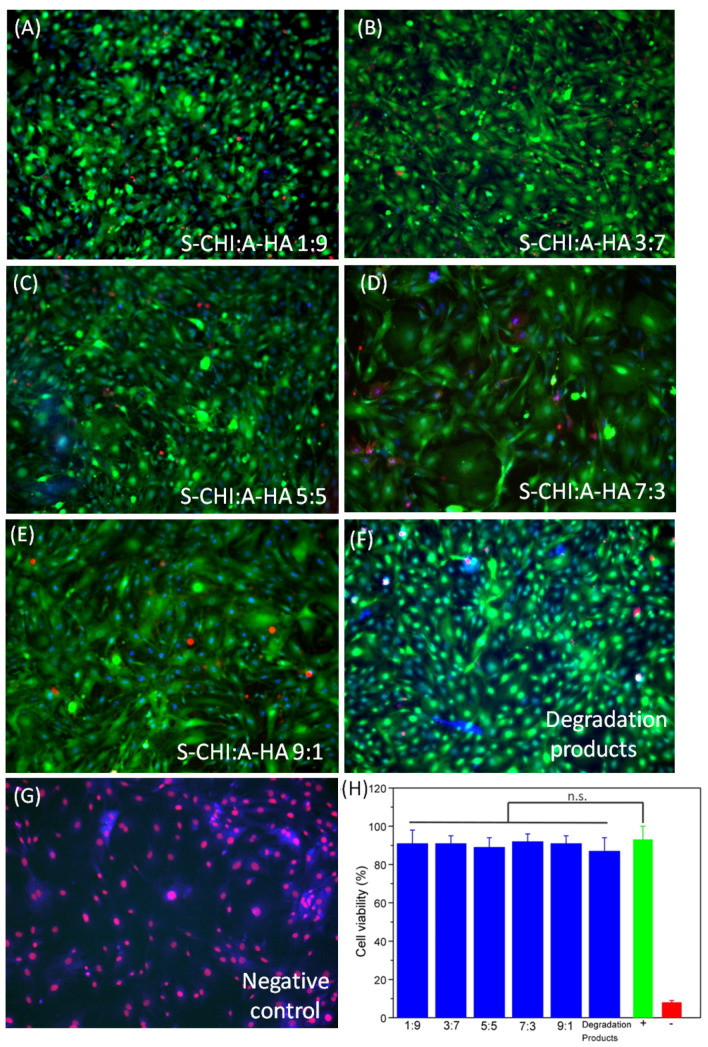
Biocompatibility of the hydrogels. Images of the cells after 24 h of contact with the hydrogels (**A**–**G**) show good biocompatibility in all tested formulations. Quantification of the ratio between dead cells with red stained nuclei and all cells (with blue nuclei), showing no statistical differences between all conditions tested (**H**). (n.s. not significant). The positive control corresponds to cell membranes incubated in ice-cold ethanol being permeable to ethidium homodimer. Cells in the negative control were cultured without hydrogel.

To determine the biocompatibility of the S-CH:A-HA hydrogels, embryonic mouse fibroblasts (MEFs) were incubated with small fragments of hydrogels and their degradation products for 24 h. Fluorescent staining was then performed to determine the percentage of cells that died due to the toxicity of the materials. The obtained data show survival values close to those of cells that were not exposed to materials or degradation products, demonstrating the excellent biocompatibility of the S-CH:A-HA gels (Figure 6).

### 3.5. Self-Healing

**Figure 7:**
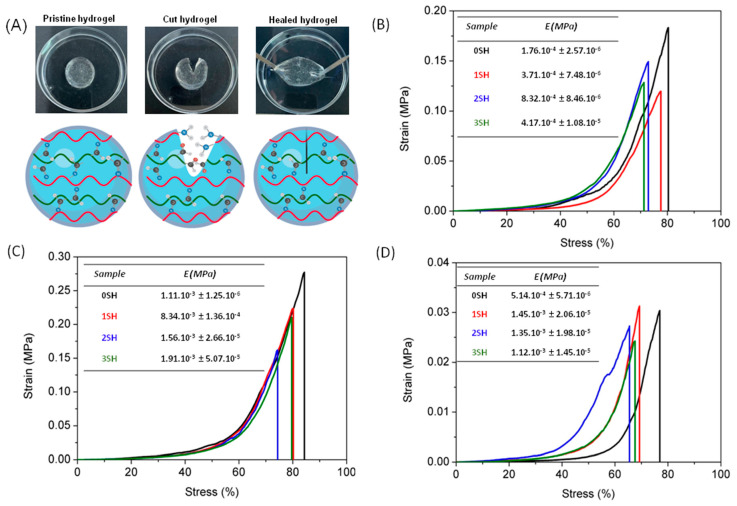
(**A**) Self-healing of 1 cut-recovery cycle. Compression strain-stress mechanical test for self-healing study of (**B**) 3:7, (**C**) 5:5 and (**D**) 7:3 S-CHI:A-HA hydrogels for 0, 1, 2, and 3 self-healing cycles (SH).

S-CHI:A-HA hydrogels have been here proposed as suitable materials for self-repairing since their gelation mechanism is based on stablishing new imine bonds (Figure 7A). Dynamic covalent bonds, such as imine, are one of the most explored mechanisms for the development of self-repairing hydrogels. This particular reversibility relies on the fact that these bonds can be disrupted and reformed autonomously through the hydrogels due to an equilibrium condition. Taking this into account, the self-healing properties of the S-CHI:A-HA 3:7, 5:5 and 7:3 hydrogels were studied and are qualitatively displayed in Figure 7B–D.

Figure 7B–D shows the variation of the mechanical properties with respect to 3 cut-recovery cycles applied to the hydrogels. Compression tests show that regardless of the number of cycles applied to the sample and its composition, the deformability of the hydrogels remains practically intact, with a small reduction between 10 and 15% in all cases. Regarding Young’s modulus, it can be seen that for 5:5 S-CHI:A-HA hydrogels, that correspond to those with the lowest swelling (Figure 7C), the moduli do not suffer variations, remaining almost constant after applying 3 cycles. However, S-CHI:A-HA 3:7 and 7:3 hydrogels (Figure 7B,D) show an increase in the Young’s moduli for the healed samples compared to pristine hydrogels, which in fact is a typical behavior of self-repairing hydrogels as a consequence of the loss of water uptake [60].

**Figure 8:**
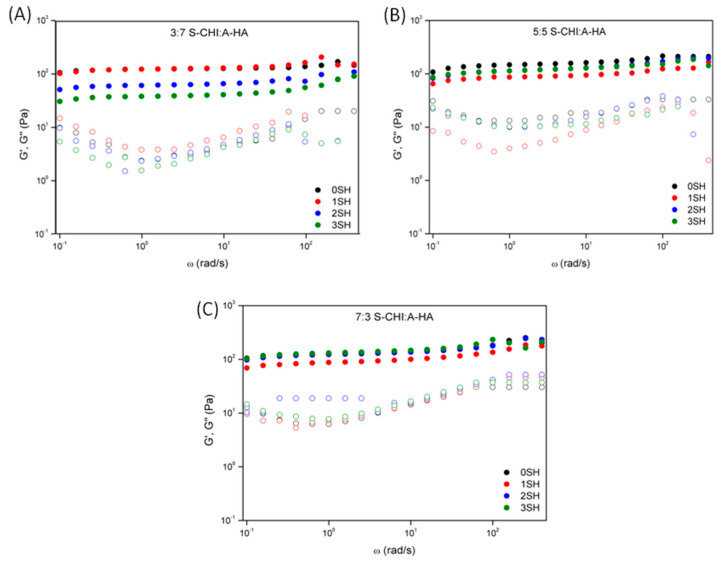
Dynamic frequency sweep for self-healing study of (**A**) 3:7, (**B**) 5:5 and (**C**) 7:3 S-CHI:A-HA hydrogels for 0, 1, 2, and 3 self-healing cycles (SH).

Further, the ability to self-repair, as well as the influence on the number of cut-recovery cycles were also studied by oscillatory rheology (Figure 8), showing that regardless of the number of self-healing cut-recovery cycles, the storage modulus (G′) was always larger than the loss modulus (G″), which is the typical behavior in which the elastic properties predominate over the viscous ones. On the other hand, results also revealed that the values of the storage moduli were quite similar for the different cut-recovery cycles, and slightly smaller compared to pristine hydrogels, which accords well with that reported for other authors [61].

These results demonstrate that these hydrogels are materials with promising characteristics due to their rapid self-healing ability (Video S1). This opens relevant possibilities for specific applications in the area of tissue regeneration and biomedicine in which biomimetic healing mechanisms and dynamics are high valuable properties.

## 4. Conclusions

The Schiff’s base reaction is a suitable method to prepared S-CHI/A-HA–based in situ-forming self-healable hydrogels. Prepared dynamic hydrogels show a clear dependence on their physico-chemical and degradation properties consistent with polysaccharides’ weight ratios. Stoichiometric ratio (5:5) increases covalent bonding, leading to the smallest swelling, the highest mechanical stability (1.1·10^−3^ MPa), and the slowest degradation profile (9 days). Similar behavior was observed for 9:1 S-CHI:A-HA hydrogels that seem to be mainly governed by CHI-CHI physical interactions. On the contrary, hydrogels with opposite stoichiometry (1:9 and 9:1, or 3:7 and 7:3) showed physico-chemical properties and degradation according to the nature of the biopolymer in excess (HA or CHI). Furthermore, all the prepared hydrogels are 100% biodegradable, however, the time required for a total disintegration varies, depending on the composition. In addition, those hydrogels with an intermediate composition (S-CHI:A-HA 3:7, 5:5 and 7:3) show a promising ability to recover from damage, being able to maintain their mechanical and rheological properties even after 3 cut-recovery cycles. Finally, all hydrogels, including their degradation products, showed good biocompatibility (>85%) that guarantees their applicability as biomaterials.

## Data Availability

The data presented in this study are available on request from the corresponding author.

## Supplementary Materials

The following supporting information can be downloaded at: https://www.mdpi.com/article/10.3390/gels8080477/s1, Video S1: Self-healing of S-CHI:A-HA 5:5 hydrogel.
